# Inactivation Kinetics of Pathogenic and Nonpathogenic Bacteria Upon In Vitro Treatment With Cold Atmospheric Pressure Plasma (CAPP)

**DOI:** 10.1155/2024/7464133

**Published:** 2024-07-22

**Authors:** Venetia Samioti, Evangelia Kriti, Aikaterini Spanou, Theofania Tsironi, Efstathios Z. Panagou

**Affiliations:** ^1^ Laboratory of Microbiology and Biotechnology of Foods Department of Food Science and Human Nutrition School of Food and Nutritional Sciences Agricultural University of Athens, Iera Odos 75, Athens GR-11855, Greece; ^2^ Laboratory of Food Process Engineering Department of Food Science and Human Nutrition School of Food and Nutritional Sciences Agricultural University of Athens, Iera Odos 75, Athens GR-11855, Greece

## Abstract

In the present study, selected pathogenic (*Salmonella* Typhimurium, *Escherichia coli*, *Pseudomonas aeruginosa*, *Listeria monocytogenes*, *Bacillus cereus*, and *Staphylococcus aureus*) and nonpathogenic (*Pseudomonas fragi*, *Pseudomonas fluorescens*, *Brochothrix thermosphacta*, *Bacillus subtilis*, *Lactiplantibacillus plantarum*, and *Leuconostoc mesenteroides*) bacteria were subjected in vitro in cold atmospheric pressure plasma (CAPP) treatment for up to 15 min and the changes in the surviving microbial population were determined. Plasma treatments were carried out by a plasma jet device, operating with argon (Ar) as carrier gas under constant flow (4.0 L/min) at a frequency of 1 MHz and an electrical voltage of 2–6 kV. Microbial inactivation data were modelled using linear and nonlinear (Geeraerd, Weibull) models, through which the corresponding kinetic parameters were calculated. After 15 min of exposure to plasma radiation, the total reduction in the bacterial populations was 2.12 log_10_ CFU mL^−1^ for *P. fragi*, 1.77 log_10_ CFU mL^−1^ for *P. fluorescens*, 2.30 log_10_ CFU mL^−1^ for *B. thermosphacta*, 1.58 log_10_ CFU mL^−1^ for *B. subtilis*, 1.31 log_10_ CFU mL^−1^ for *L. plantarum*, 3.80 log_10_ CFU mL^−1^ for *L. mesenteroides* (highest reduction observed), 1.12 log_10_ CFU mL^−1^ for *S*. Typhimurium, 1.18 log_10_ CFU mL^−1^ for *E. coli*, 1.43 log_10_ CFU mL^−1^ for *L. monocytogenes*, 1.32 log_10_ CFU mL^−1^ for *B. cereus*, 0.88 log_10_ CFU mL^−1^ for *S. aureus*, and 0.73 log_10_ CFU mL^−1^ for *P. aeruginosa*. The results showed a higher reduction in the population of nonpathogenic microorganisms compared to pathogens. The relatively small decrease in the inactivation of bacteria indicates that parameter optimization is necessary to be considered to improve the efficacy of the treatment.

## 1. Introduction

Cold atmospheric pressure plasma (CAPP) is an emerging novel technology that is extensively used in engineering, microelectronics, and materials science, and during the last years, it has received attention in new fields such as medicine, agriculture, and the food industry [1, 2]. At the same time, potential combinations of cold plasma with other innovative nonthermal technologies, such as pulsed electric fields, pulsed light, ultrasounds, and nanotechnology applications, including the use of nanoemulsions and nanoparticles, have been also reported [3].

Plasma is defined as the fourth state of matter. It can be considered as a partially ionized gas that consists of electrons, ions, neutral atoms, and molecules and also free radicals, excited species, and photons. Nonthermal plasma (cold plasma) is known as nonequilibrium plasma, as it exhibits electron temperature of some 10,000 K, while the gas temperature can be close to ambient [4]. Cold plasmas can be generated either in low or atmospheric pressure using numerous gases or combinations (e.g., air, oxygen, nitrogen, helium, argon (Ar), and precursors). The generation of CAPP can be carried out by various types of devices like plasma jets, dielectric barrier discharge (DBD), glow discharge, and corona discharge, by using different types of sources like sinusoidal kilohertz (kHz) AC, DC, pulsed DC, radio frequency (RF), and microwave (MW) frequency [5].

Plasma inactivates microorganisms via three primary mechanisms: (a) UV irradiation of cell DNA, (b) UV irradiation of cell membrane and intracellular components, and (c) chemical interaction with charged particles and reactive oxygen-nitrogen species (RONS) [6]. Though the most efficient microbe inactivation comes from a combination of the above mechanisms, it is stated that the inactivation process is mainly controlled by the RONS. These plasma reactive species created in the gas phase include O, O_3_, OH, NO, etc., and when they come into contact with the surface of bacteria, they induce several other inactivation processes, such as the oxidation of macromolecules and membrane erosion [7].

In the food sector, this technology has been extensively studied in the last 10 years, mainly due to the shift towards the application of environmentally friendly, cost and energy efficient, minimal processing methods for the production of safe and high-quality foods with extended shelf life, without affecting their nutritional value and sensory properties, as compared to conventional thermal processing [8, 9]. As cold plasma is an effective method for the inactivation of a broad spectrum of microorganisms, including bacteria, yeasts, and moulds, it can be effectively used for the disinfection of fresh products, such as fruits and vegetables. Fresh products are extremely fragile and constitute one of the main sources of food waste worldwide. The use of CAPP can be expanded to mycotoxin detoxification. For instance, aflatoxins can be found in a wide range of nuts. These carcinogenic agents can be significantly reduced by the exposure of cold plasma. The properties of this process make it also appropriate for the sterilization of food packaging material, like plastic bottles, lids, and films, in a safe and instant way. In addition, low temperature plasma treatment provides significant possibilities especially for the decontamination of heat sensitive materials, for example, polythene ethylene and polycarbonate [1, 10, 11].

Although CAPP can be an effective solution to the above-mentioned issues, it has not found extensive application on an industrial scale. Specifically, the application of plasma-treated water (PTW) in an industrial washing process on leafy greens has been recently reported [12]. The PTW application on mixed lettuce increased the reduction of microbial load up to 2 log units on the product itself and on the wash water compared to the application of standard washing, by mainly reducing the ability of proliferation and affecting membrane integrity of microorganisms. In addition, Kilonzo-Nthenge et al. [13] studied the efficacy of a DBD CAPP system against *Salmonella* and *Escherichia coli* on the surface of Golden Delicious apples after spot inoculation with a two-strain mixture at 8 log_10_ CFU mL^−1^ on their surface and reported a decrease higher than 5.0 log_10_ CFU/cm^2^ in both bacteria after 180 and 240 s exposure time. Mošovská et al. [14] investigated the inactivation of *E. coli*, *Salmonella* Enteritidis, and *Bacillus subtilis* treated with a multihollow surface DBD plasma using different working gases (ambient air, O_2_, and N_2_) and reported that all plasma treatments inactivated tested microorganisms, depending on the working gas employed.

The aim of this study was to evaluate in vitro the effect of CAPP on the kinetic behavior of selected pathogenic and nonpathogenic microorganisms and to estimate the kinetic parameters for bacterial cell inactivation by means of linear and nonlinear inactivation models.

## 2. Materials and Methods

### 2.1. Bacterial Species and Inoculum Preparation

*Salmonella* Typhimurium, *E. coli*, *Pseudomonas aeruginosa*, *Listeria monocytogenes*, *Bacillus cereus*, *Staphylococcus aureus*, *Pseudomonas fragi*, *Pseudomonas fluorescens*, *Brochothrix thermosphacta*, *B. subtilis*, *Lactiplantibacillus plantarum*, and *Leuconostoc mesenteroides* from the collection of the Laboratory of Microbiology and Biotechnology of Foods of the Agricultural University of Athens (Greece) were used in this study. Stock cultures were maintained in vials of treated beads in a cryoprotective fluid (Protect Bacterial Preservers, Technical Service Consultants Ltd, Lancashire, United Kingdom) at −80°C until use. The cultures were revived by adding one bead to 9 mL of tryptic soy broth (TSB; 4021552, Biolife, Milan, Italy) and incubating the cultures for 18–24 h at 37°C for pathogenic bacteria, 30°C for *B. subtilis*, and 25°C for the other microorganisms. In the case of lactic acid bacteria (LAB), inoculation was undertaken in de Man–Rogosa–Sharpe broth medium (MRS Broth; 4017292, Biolife, Milan, Italy) for 48–72 h at 30°C. For experiments, 100 *μ*L of each bacterial suspension was transferred into 9 mL of TSB or MRS broth and incubated for 18 h at the same temperature conditions. Bacterial cells were harvested by centrifugation at 7000 × g for 10 min at 4°C. The resulting pellet was washed with sterile Ringer's solution (LAB M Limited, Lancashire, United Kingdom), recentrifuged, and resuspended in the same diluent to a final volume of 10 mL. Finally, the cells were serially diluted in sterilized Ringer's solution to obtain a final inoculum of ca. 8.0–9.0 log CFU mL^−1^.

### 2.2. Inoculation of Petri Dishes and Plasma Treatment

In order to quantify the effects of cold plasma treatment on the survival of the selected microorganisms, 1 mL from each bacterial suspension was serially diluted in 9 mL of sterilized Ringer's solution. Afterwards, a 0.1 mL volume of bacterial suspension from each dilution was surface plated in duplicate on de Man–Rogosa–Sharpe agar (MRS agar; 4017282, Biolife, Milan, Italy) and tryptic soy agar (TSA; 4021502, Biolife, Milan, Italy) for LAB and for the other bacteria, respectively. Each Petri dish was labelled according to the dilution factor and plasma exposure time.

**Figure 1:**
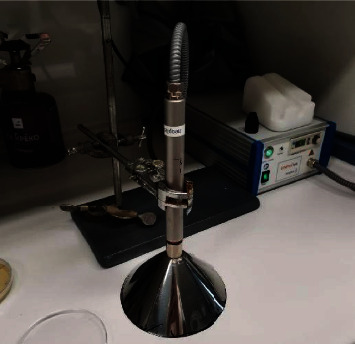
The CAPP jet system device (kINPen® IND) with the metallic conical end in operation.

The device used to produce the CAPP was the kINPen® IND jet system device (neoplas GmbH, Germany), consisting of a pen-sized hand-held unit for plasma generation at atmospheric pressure, a power supply, and a gas supply unit. The plasma jet was generated at the tip of a pin-type electrode (1 mm diameter) that was surrounded by a dielectric capillary and a grounded electrode. The distance between the tip of the electrode and the nozzle was 3.5 mm, while the inner and outer diameter of the dielectric capillary was 1.6 mm and 2 mm, respectively. Operating at a sinusoidal frequency equal to 1 MHz, the system power was about 20 W, while the power loss was less than 3.5 W for voltage values of 2–6 kV. In addition, the gas flow rate was 4 L per minute (standard liters per minute, slm, according to information provided by the manufacturer). The system was operated by supplying Ar as carrier gas. For uniform distribution of the plasma over the entire surface of the Petri dish, a metallic conical end (Ø 10 cm) was adjusted to the jet nozzle maintaining a standard distance of 65 mm between the plasma source and the sample (Figure 1). The selected bacteria were exposed to CAPP for 3, 6, 9, 12, and 15 min.

### 2.3. Incubation and Enumeration of Surviving Cells

After the treatment with plasma, inoculated Petri dishes were incubated in high precision incubation chambers (MIR-153, Sanyo Electric Co., Osaka, Japan) at the appropriate temperature and time. Specifically, *P. fragi*, *P. fluorescens*, and *B. thermosphacta* were incubated at 25°C for 48 h. *B. subtilis*, *L. plantarum*, and *L. mesenteroides* were incubated at 30°C for 48 h. In addition, *S.* Typhimurium, *P. aeruginosa*, *E. coli*, *B. cereus*, *L. monocytogenes*, and *S. aureus* were incubated at 37°C for 24 h. The colonies were counted after incubation, and the effect of CAPP was determined by the log reduction of bacterial cells comparatively to the initial microbial population. Data from plate counts were log transformed prior to further analysis and expressed as log_10_ CFU mL^−1^ ± standard deviation. The experiment was repeated twice with duplicate plates analyzed for each sampling point (*n* = 4).

### 2.4. Determination of Inactivation Parameters

Inactivation curves were constructed by plotting survival bacterial cells against CAPP treatment time. Different models (log-linear, Geeraerd, and Weibull) were fitted to the inactivation data using GInaFiT software (Ver. 1.7 for Microsoft Excel) to determine the kinetic parameters and describe the inactivation curves due to CAPP treatment.

*Τ*he log-linear model [15] was fitted to the inactivation data, as shown in Equation (1):

(1) $$\begin{array}{c} \log_{10}N\left(t\right)=\log_{10}N_{0}-k_{\max}∙t\; \end{array}$$

where *N*(*t*) is the population (log_10_ CFU mL^−1^) at time *t* (second), *N*_0_ is the initial population (log_10_ CFU mL^−1^), and *k*_max_ is the maximum specific inactivation rate (1/second). This model is based on the hypothesis that all cells in the microbial population have the same sensitivity and resistance to stress. However, these conditions cannot be fulfilled, as within a microbial population, there is no absolute uniformity between cells. The diversity of bacterial cells is most pronounced in cases where more innovative and milder processing methods are used to control bacteria. As a consequence, deviations from linearity can be observed and the use of nonlinear models becomes necessary.

The Geeraerd model [16] was also fitted to the counts of surviving bacterial cells, as shown in Equation (2):

(2) $$\begin{array}{c} N\left(t\right)=\left(N_{0}-N_{\text{res}}\right)∙e^{-k_{\max}∙t}∙\frac{e^{k_{\max}∙t_{s}}}{1+\left(\left(e^{-k_{\max}∙t_{s}}-1\right)∙e^{-k_{\max}∙t}\right)}+N_{\text{res}} \end{array}$$

where *t*_*s*_ is the duration of the shoulder (second) and *k*_max_ is the maximum specific inactivation rate (1/second), and *N*_0_ and *N*_res_ are the initial and residual populations (log_10_ CFU mL^−1^), respectively. This mathematical model includes the shoulder and tailing configurations, taking into account the delay to the beginning of the log-linear inactivation phase and the corresponding formation of a “tail” after the linear part of the curve.

The Weibull model [17] was the third model employed, as shown in Equation (3):

(3) $$\begin{array}{c} \frac{N\left(t\right)}{N_{0}}=10^{-{\left(t/\delta \right)}^{p}} \end{array}$$

where *δ* is a scale parameter that denotes the time (second) for the first decimal reduction and *p* is the shape factor of the curve. For *p* > 1, concave-downward curves are obtained, whereas for *p* < 1, concave-upward curves are described. In using this model, it is assumed that the survival of bacterial cells follows the Weibull distribution. In the event that the survival curve presents tailing, the modified Weibull model was fitted to the experimental data, which is defined by Equation (4):

(4) $$\begin{array}{c} N\left(t\right)=\left(N_{0}-N\left(t\right)\right)∙10^{-{\left(t/\delta \right)}^{p}}+N_{\text{res}} \end{array}$$

where *N*_res_ is the residual population (log_10_ CFU mL^−1^).

### 2.5. Statistical Analysis

Statistical analysis was performed by one-way analysis of variance (ANOVA) using Stata 18 software (StataCorp. 2023, Stata Statistical Software: Release 18; College Station, TX: StataCorp LLC.) for the kinetic parameters of *k*_max_ (*n* = 4) of the log-linear and Geeraerd models and for *δ* (*n* = 4) of the Weibull models. Significant differences were determined by Tukey's HSD multiple range test (*p* < 0.05).

## 3. Results and Discussion

### 3.1. Effect of Plasma Treatment on the Inactivation of Selected Bacteria

**Figure 2:**
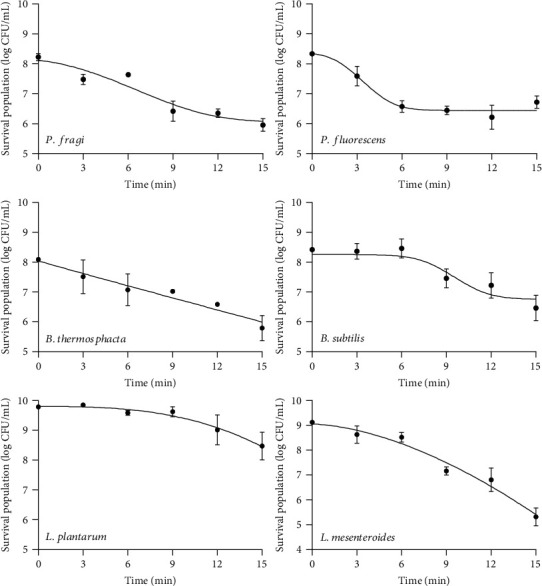
Inactivation curves of *P. fragi*, *P. fluorescens*, *B. thermosphacta*, *B. subtilis*, *L. plantarum*, and *L. mesenteroides* during CAPP treatment for 15 min. Lines were fitted with the log-linear, Geeraerd, and Weibull models. The error bars represent the standard deviation of measurements of two plates in two separate experiments (*n* = 4).

**Figure 3:**
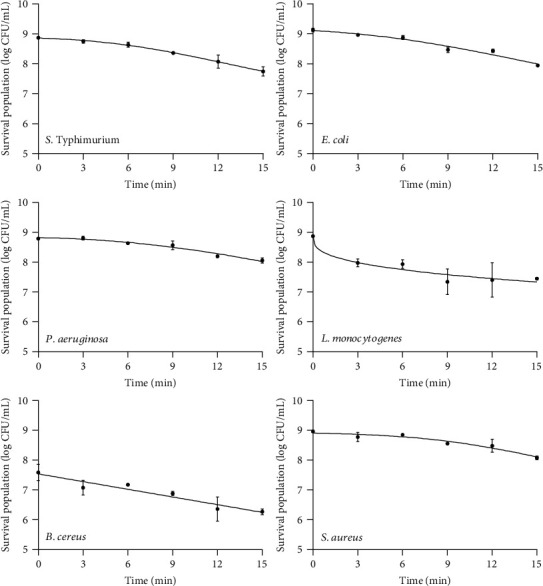
Inactivation curves of *S.* Typhimurium, *E. coli*, *P. aeruginosa*, *L. monocytogenes*, *B. cereus*, and *S. aureus* during CAPP treatment for 15 min. Lines were fitted with the log-linear, Geeraerd, and Weibull models. The error bars represent the standard deviation of measurements of two plates in two separate experiments (*n* = 4).

The inactivation profile of all microorganisms presented variation in sensitivity to CAPP treatment as illustrated in Figures 2 and 3 for nonpathogenic and pathogenic bacteria, respectively. Specifically, *P. fragi* showed a bacterial load reduction of 2.12 log_10_ CFU mL^−1^, with most reductions taking place in the first 9 min of plasma treatment. A similar pattern was observed for *P. fluorescens* that presented 1.77 log_10_ CFU mL^−1^ reduction mainly in the first 6 min of plasma treatment. The bacterium *B. thermosphacta* showed also a population reduction of 2.3 log_10_ CFU mL^−1^ after 15 min of plasma exposure. Further on, *B. subtilis* showed an overall decrease of 1.58 log_10_ CFU mL^−1^. This microorganism initially presented relative resistance to the plasma application without a noticeable reduction in its population, at least until the first 8 min of plasma exposure. The lactic acid bacterium *L. mesenteroides* showed the highest reduction compared to the other microorganisms investigated. Specifically, the initial population of this bacterium prior to plasma treatment was 9.12 log_10_ CFU mL^−1^, and after 15 min of exposure to CAPP, it decreased by 3.80 log_10_ CFU mL^−1^. Finally, *L. plantarum* showed the second smallest population reduction among the nonpathogenic microorganisms (1.31 log_10_ CFU mL^−1^) and the longest shoulder duration (10 min) compared to all microorganisms tested.

Regarding the pathogenic bacteria, the CAPP treatment reduced the initial population of *S*. Typhimurium by 1.12 log_10_ CFU mL^−1^ after 15 min of exposure. A similar inactivation pattern was observed for *E. coli*, with counts decreasing from 9.13 to 7.94 log_10_ CFU mL^−1^ (i.e., by 1.18 log_10_ CFU mL^−1^). It needs to be noted that *P. aeruginosa* presented the lowest population reduction of all microorganisms assayed (0.73 log_10_ CFU mL^−1^), even after 15 min of exposure to plasma. The bacterium *L. monocytogenes* showed the greatest decrease in its population among the pathogens. In particular, its bacterial load decreased from the initial value of 8.87 log_10_ CFU mL^−1^ by 1.43 logs in the first 9 min of exposure. The second largest population reduction among the pathogens tested was observed for *B. cereus*, with a population decrease in the order of 1.32 log_10_ CFU mL^−1^. Finally, the reduction in the population of *S. aureus* was also small (only 0.88 log_10_ CFU mL^−1^), as shown in Figure 3, ranking it as the second microorganism in terms of the lowest population reduction by plasma application.

Overall, the bacterial population reduction exhibited by each microorganism ranged from 0.73 to 3.80 log_10_ CFU mL^−1^. Notably, nonpathogenic microorganisms presented higher level of reduction and seemed to be more sensitive to the application of CAPP compared to pathogenic bacteria. Many researchers have investigated the inactivation effects of CAPP on both spoilage and pathogenic bacteria, as well as on bacteria with different cell characteristics [13, 18–24]. The carrier gas type has a determinant influence on the kind and also the concentration of reactive species in cold plasma. In particular, the use of oxygen, nitrogen, or ambient air as a carrier gas results in the formation of oxygen and nitrogen reactive species. On the other hand, inert gases, such as Ar or helium, offer the possibility of easier ionization and consequently the production of higher quantities of electrons, excited molecules, and atoms [25]. Other process parameters, such as discharge type, power, excitation frequency, gas flow rate, exposure time, and distance between the discharge and target, have a significant impact on the output of exposure to plasma. Noriega et al. [26] reported higher inactivation of *Listeria innocua* on chicken meat and chicken skin by optimizing some of the above-mentioned operating conditions. In particular, they investigated the effect of higher voltage values, excitation frequency, and the use of a helium–oxygen flow and reported higher than 1 and 3 log reductions on chicken skin and muscle after 8 and 4 min treatment, respectively, concluding that the efficacy of plasma treatment is affected by surface topography.

Reactive species are an important aspect of the effect of cold plasma on bacterial cells. As a consequence, the carrier gas type contributes crucially to the inactivation rate of each microorganism. Olatunde, Benjakul, and Vongkamjan [21] reported that after 5 min of plasma treatment, all tested bacteria (*P. aeruginosa*, *E. coli*, *Vibrio parahaemolyticus*, *L. monocytogenes*, and *S. aureus*), with an initial load of 6 log_10_ CFU mL^−1^, were not detected. Also, according to Zhao et al. [24], no colonies of *P. aeruginosa* were detected after 5 or 9 min of CAPP treatment, depending on the gas used for plasma generation (air or nitrogen). The results of the above-mentioned researchers are not in full agreement with the observations reported in this work, as the same bacteria were more resistant to the applied plasma treatment. This difference could be attributed to the working gas employed in plasma generation, as Olatunde, Benjakul, and Vongkamjan [21] exposed bacteria to plasma produced from Ar/oxygen mixture and Zhao et al. [24] used air or nitrogen as a carrier gas. Additionally, according to Mošovská et al. [14], *E. coli* and *B. subtilis* were also sensitive to plasma treatment based on ambient air, nitrogen, and oxygen, as their populations reduced significantly within seconds. Specifically, the use of ambient air as a working gas caused a reduction of 4.70 log_10_ CFU mL^−1^ in 15 s and 3.59 log_10_ CFU mL^−1^ in 25 s for *E. coli* and *B. subtilis*, respectively. Short treatment times (up to 140 s) were also reported by Huang et al. [19] for *S.* Typhimurium and *S. aureus* populations on solid surfaces and liquid suspensions during DBD cold plasma treatment generated by an oxygen/nitrogen mixture.

The efficacy of cold plasma produced from ambient air is associated with the presence of oxygen and air humidity, which result in the formation of very active ROS and RNS. Another important aspect affecting the efficacy of plasma treatment is the device and the discharge type used. Specifically, previous researchers [14, 19, 21] used DBD devices, a simple way for generating cold plasma, in which the discharge area is not separated from the plasma and target interaction region. Especially, the new geometry of multihollow surface DBD plasma employed by Mošovská et al. [14] was proved to be very effective, as it achieved significant inactivation in very short time. Another significant parameter enhancing the efficacy of the plasma treatment was the distance between the samples and the ceramics with the embedded electrodes, which was kept to 1 mm. On the other hand, Zhao et al. [24] applied a cold plasma jet treatment to an initial population of 8.48 log_10_ CFU mL^−1^*P. aeruginosa* at a distance of 10 cm. Considering that the parameters of the above-mentioned research were similar to those reported in the present study, in terms of microorganism, initial population, plasma device, and exposure distance, it could be assumed that the most determining factor affecting inactivation was the carrier gas employed in plasma generation. Indeed, oxygen, nitrogen, or ambient air was more effective than Ar in the inactivation of this bacterium. However, other factors should not be neglected, such as the different frequency and electrical voltage of the plasma device that may have contributed to the discrepancy of the results. Additionally, concerning the bacterial structure, Gram-positive bacteria are generally more resistant to plasma treatment duo to their thicker peptidoglycan layer, which protect them from the plasma radiation [5]. On the other hand, Gram-negative strains have a thin peptidoglycan layer with an outer membrane, consisting of lipopolysaccharides and phospholipids. As a result, the cell wall can be injured by exposure to plasma to a greater extent in an irreversible way. A study conducted to examine the mechanism of bacterial inactivation by Olatunde, Benjakul, and Vongkamjan [21], using cell wall integrity and DNA damage as indicators, showed differences between positive and negative Gram bacteria. After scanning electron microscope (SEM) analysis, it was found that Gram-negative microorganisms showed the most significant visible cellular damage.

### 3.2. Modelling Inactivation Kinetics

The mathematical models used to fit the experimental data described satisfactorily the population dynamics of the surveyed microorganisms as a function of CAPP treatment (Figures 2 and 3). In addition, the kinetic parameters of the models are estimated and presented in Table 1. The shape of the curves differs considerably for each microorganism, due to their different resistance to plasma treatment. The low values of the root mean squared error (RMSE) and the high values of the coefficient of determination (*R*^2^ > 0.92) indicate the good fit of the models to the experimental data.

For the experimental data obtained from the inactivation of *L. plantarum* and *S. aureus*, the model of Geeraerd provided a very good fit, as demonstrated by the respective values of RMSE and *R*^2^. Both bacteria showed a long-term shoulder phase that could be attributed to the initial resistance of the bacterial cells. The maximum specific inactivation rate (*k*_max_) is also a crucial parameter in modelling the death of microorganisms, as it provides important information about the efficiency of the process. The value of this parameter for *L. plantarum* (0.53 min^−1^) indicates a more intense effect (i.e., faster inactivation) of plasma treatment, compared to *S. aureus* (0.29 min^−1^), even though a nonstatistically significant difference (*p* < 0.05) was found among the *k*_max_ values. This could be attributed to the fact that *L. plantarum* is a rod-shaped bacterium, generally 0.9–1.2 *μ*m wide and 3–8 *μ*m long. On the other hand, *S. aureus* is a spherical bacterium with a diameter of approximately 0.5–1.5 *μ*m. The spherical structure of cocci can often contribute their resistance to stress, due to their reduced surface area and the limited inflow of harmful compounds [27, 28]. Thus, the efficiency of plasma treatment on these microorganisms could be affected by the shape of the bacterium. For the inactivation of *P. fragi*, the Geeraerd model with a “tail' effect presented the best fit to the experimental data. The maximum specific inactivation rate (*k*_max_) was 0.56 min^−1^, and the short duration of the shoulder phase indicated that inactivation started in the first few minutes of exposure to CAPP.

Further on, the Weibull model was better fitted to the inactivation data for *L. mesenteroides*, *E. coli*, *P. aeruginosa*, *L. monocytogenes*, and *S.* Typhimurium, as can be inferred from the performance of the relevant indices (*R*^2^ > 0.94, RMSE < 0.31). The value of the shape factor (*p*) was above 1, indicating downward concavity in the survival curves, with the exception of *L. monocytogenes* where the shape factor was less than 1, indicating upward concavity in the survival curve of the pathogen. A link has been attempted between the values of the shape factor (*p*) with microbial inactivation to provide a biological insight *ο*n the use of the Weibull model. Thus, values of *p* < 1 indicate that the remaining cells are adapting to the stress and have less probability of dying. In contrast, *p* > 1 indicates that the remaining cells are becoming increasingly susceptible to the stress factor and have lower probability of survival due to cumulative damage occurred [29]. The scale parameter *δ* that denotes the time for the first decimal reduction varied from a minimum value of 4.25 min for *L. monocytogenes* to a maximum value of 17.02 min for *P. aeruginosa*. Statistical analysis indicated that there were significant differences (*p* < 0.05) of the *δ* values of *L. monocytogenes* and *L. mesenteroides*, compared to those of *E. coli*, *P. aeruginosa*, and *S.* Typhimurium, indicating a significantly faster first decimal reduction of the first-mentioned microorganisms (ranging from 4.25 to 6.91 min) as opposed to the latter (ranging from 13.93 to 17.02 min).

The inactivation kinetics of *P. fluorescens* and *B. subtilis* were also described by using the modified Weibull model taking into account the “tail” effect that represents the part of the bacterial population that is more resistant to CAPP treatment. The residual populations (*N*_res_) were similar, with values of 6.44 and 6.77 log_10_ CFU mL^−1^ for *P. fluorescens* and *B. subtilis*, respectively. The presence of tailing could be attributed to the variability of sensitivities to the lethal agent in the bacterial population, or adaptation to the stress that makes the remaining cells more resistant [28]. Even in the case of a pure culture, as in this study, biological heterogeneity among the cells results in subpopulations with individual inactivation kinetics. Some cells have the ability to adapt to the applied stress as opposed to others that are more vulnerable, and they are inactivated from the early stages of processing. As a result, the inactivation kinetics does not always follow first-order approach, and the curve may deviate from linearity. Some factors that affect the behavior of bacterial cells are the age, the physiological state, and the phenotypic or genotypic variations. Additionally, this phenomenon can be attributed to deviations of the biochemical and physiological characteristics [30]. Thus, the model with the best fit to the experimental data must be selected every time in order to describe accurately the inactivation pattern. Consequently, inactivation curves should be regarded as the cumulative form of underlying distribution of individual inactivation times, resulting in deviations from linearity with the presence of shoulder and/or tailing [29]. By comparing the values of *δ*, representing the time for the first decimal reduction, a significant difference (*p* < 0.05) was observed among them, and it can be concluded that *P. fluorescens* was more sensitive to CAPP treatment (*δ* = 3.53 min), followed by *B. subtilis* (*δ* = 10.12 min). Notably, the populations of the latter two bacteria remained almost unchanged during CAPP treatment for 6 min prior to inactivation.

Finally, the survival curves of *B. thermosphacta* and *B. cereus* presented first-order inactivation kinetics. The inactivation of both bacteria started from the first minute of exposure to CAPP and continued at a steady rate. The presence of linear inactivation kinetics for these bacteria could be attributed to the assumption that the cells of the population are homogeneous with identical inactivation times and hence follow a linear trend. Considering the *k*_max_ values (0.31 min^−1^ for *B. thermosphacta* and 0.20 min^−1^ for *B. cereus*), it can be concluded that *B. thermosphacta* was significantly (*p* < 0.05) more sensitive to CAPP treatment compared to *B. cereus*.

## 4. Conclusions

In this study, the effect of CAPP on the in vitro inactivation of 12 bacteria was investigated, including 6 major food pathogens and 6 nonpathogens with spoilage potential. The level of population reduction varied depending on the bacterium under study and ranged from 0.73 to 3.80 log_10_ CFU mL^−1^ after 15 min of plasma treatment. Generally, pathogenic bacteria seemed to be more resistant to CAPP treatment based on the number of logarithmic reductions in their population. All bacteria presented nonlinear inactivation curves, with the exception of *B. thermosphacta* and *B. cereus*, which followed first-order kinetics. Finally, the presence of tailing in the inactivation curves of *P. fragi*, *P. fluorescence*, and *B. subtilis* indicates adaptation of the survival cells to the applied CAPP. Further research is necessary to optimize the parameters of CAPP treatment, as, for example, using a different gas, or combining Ar with oxygen or ambient air to increase the amount of reactive species within CAPP, change the gas flow rate, to improve the antimicrobial efficacy of CAPP against pathogenic and spoilage bacteria.

## Figures and Tables

**Table 1:** Parameter estimation and statistical indices of the different models used for fitting the experimental data.

**Model type**	**N**_0_ **(log**_**10**_**CFU mL**^**−1**^**)**	**N**_**r** **e** **s**_ **(log**_**10**_**CFU mL**^**−1**^**)**	**k**_max_ **(min**^**−1**^**)**	**t**_**s**_ **(min)**	**δ** **(min)**	**p**	**RMSE**	**R** ^2^
Geeraerd								
*L. plantarum*	9.79 ± 0.08	—	0.53 ± 0.10^a^	9.27 ± 1.07			0.11	0.97
*S. aureus*	8.91 ± 0.08	—	0.29 ± 0.09^a^	9.19 ± 2.03			0.09	0.95
*P. fragi*	8.12 ± 0.39	6.03 ± 0.51	0.56 ± 0.41^a^	2.44 ± 4.41			0.40	0.92
Weibull								
*L. mesenteroides*	9.05 ± 0.27	—			6.91 ± 1.42^a^	1.67 ± 0.41	0.31	0.97
*E. coli*	9.11 ± 0.10	—			13.93 ± 1.19^b^	1.48 ± 0.42	0.11	0.96
*P. aeruginosa*	8.81 ± 0.06	—			17.02 ± 1.12^b^	1.80 ± 0.47	0.07	0.97
*L. monocytogenes*	8.88 ± 0.19	—			4.25 ± 2.81^a^	0.34 ± 0.15	0.19	0.94
*S.* Typhimurium	8.86 ± 0.02	—			13.96 ± 0.26^b^	1.61 ± 0.11	0.03	0.99
Weibull tailing								
*P. fluorescens*	8.34 ± 0.12	6.44 ± 0.07			3.53 ± 0.41^a^	1.61 ± 0.41	0.12	0.99
*B. subtilis*	8.27 ± 0.07	6.77 ± 0.10			10.12 ± 0.44^b^	3.90 ± 0.98	0.10	0.99
Log-linear								
*B. thermosphacta*	8.04 ± 0.14	—	0.31 ± 0.04^b^				0.20	0.95
*B. cereus*	7.54 ± 0.12	—	0.20 ± 0.03^a^				0.16	0.92

*Note: k*_max_ and *δ* values with different superscripts were significantly different (*p* < 0.05) as shown by Tukey's HSD multiple range test. Different letters indicate differences among data of each model (*p* < 0.05). The results are expressed as the mean ± standard error of four replicates.

## Data Availability

The original contributions presented in the current study are available from the corresponding author upon request.
